# American Medical Association

**Published:** 1862-04

**Authors:** 


### AMERICAN MEDICAL ASSOCIATION.

We think there has been no time in the history of the country, when our Medical Societies, both local and general, could be more useful than at present. We are glad to see that such State Societies as have recently held their annual meetings, have been well attended. For ourselves, we can say to our brethren elsewhere, that we shall greet them in our goodly city with the greatest pleasure. We trust the proper notices will be issued by the Secretaries and Committee of Arrangements, without delay.—*Med. Examiner.*

*American Medical Association.*—The delegates to this Association from this city, upon consultation, have come to the conclusion to advise that no meeting of the Association be held at Chicago the present year, as the Southern States would not probably be represented. We are pleased to find our position upon the subject so emphatically indorsed.—*Medical and Surgical Reporter.*

